# A COM-B analysis of facilitators of and barriers to smoking cessation among Chinese smokers: A qualitative study

**DOI:** 10.18332/tid/174128

**Published:** 2023-11-16

**Authors:** Xiaoyun Xie, Yi Nan, Bingliang Lin, Tianqi Chen, Luge Zhang, Lin Xiao

**Affiliations:** 1 Tobacco Control Office, Chinese Center for Disease Control and Prevention, Beijing, People’s Republic of China; 2 School of Public Health, The University of Hong Kong, Hong Kong SAR, People’s Republic of China

**Keywords:** qualitative research, smoking cessation, Chinese, COM-B

## Abstract

**INTRODUCTION:**

Smoking prevalence remains high in China with a low cessation motivation level, despite the government’s tobacco control efforts. There is a lack of research specifically examining perceptions, attitudes, and behaviors related to smoking cessation in this region, particularly from a theory-based deductive perspective. Utilizing the COM-B (Capability, Opportunity, Motivation-Behavior) model as a theoretical framework, this study aimed to identify facilitators and barriers to smoking cessation among Chinese smokers.

**METHODS:**

The study employed semi-structured individual interviews with 40 participants. Each interview spanned approximately 30 minutes. The participants, constituting both current and former smokers, were all aged ≥18 years (n=40). Interview data were then examined using a directed content analysis approach.

**RESULTS:**

Analysis revealed three interrelated themes. Capability: Smokers face challenges when resisting peer pressure and dealing with life after quitting. They also lack knowledge about smoking, quitting techniques, and withdrawal symptoms. Opportunity: Changing societal attitudes towards smoking create opportunities for quitting, but these are hindered by inadequate cessation services and a lack of family support. Motivation: Smokers' motivation to quit is mainly driven by health concerns. Resistance to quitting often stems from the belief that smoking is a personal choice or just a habit. Excessive emphasis on willpower may hinder motivation to quit.

**CONCLUSIONS:**

To enhance smoking cessation efforts in China, three key aspects should be considered: capability, opportunity, and motivation. Publicity and educational campaigns should target common misconceptions about smoking as a personal freedom, correct the overemphasis on willpower, and widely promote available cessation services. A crucial aspect is shifting societal norms to foster anti-smoking sentiments. Effective strategies may involve using real-life stories to illustrate smoking's health consequences, disseminating information about cessation services in maternity centers, enhancing services through mobile health initiatives, and empowering families to support smokers in their quit attempts.

## INTRODUCTION

Smoking has remained a critical global public health issue for several decades, with China shouldering a significant share of the burden; currently, 308 million adult Chinese citizens are active smokers^1^. Data from the Global Burden of Disease (GBD) indicated that in 2019, an estimated 2.4 million deaths in China were directly attributed to smoking^2^. Furthermore, non-smokers are also at substantial health risk due to secondhand smoke exposure; according to the global adult tobacco survey (GATS) conducted in 2018, approximately 50.9% of participants in China working in indoor environments were exposed to tobacco smoke at their workplace^1^. Moreover, a macroeconomic study projected that from 2015 to 2030, the costs associated with tobacco-attributable non-communicable diseases (NCDs) could reach a staggering total of $2.3 trillion in China^3^.

Reducing the health burden associated with smoking necessitates guiding Chinese smokers towards cessation, and providing motivation and support for this process. Despite endeavors by the Chinese government to control the tobacco epidemic in the country, such as the ratification of the World Health Organization Framework Convention on Tobacco Control (WHO FCTC) in 2005^4^, which advocates for assistance in tobacco cessation, and incorporating tobacco control into the 4th prioritized action of ‘Healthy China Initiative 2019–2030’ ^5^, the prevalence of smoking remains high. Furthermore, the motivation to quit rests at an undesirable level: only 5.6% of current smokers intended to quit within the next month in 2018^1^. Compounding this predicament is the entrenched tendency among Chinese smokers to rely on personal willpower rather than professionally administered cessation assistance^1^, as study suggested that only 2–5% of untreated smokers can maintain long-term abstinence^6^. Consequently, understanding the quit intentions amongst Chinese smokers and identifying the factors linked to cessation, should be of paramount importance.

The COM-B (Capability, Opportunity, Motivation-Behavior) Model^7^ posits that behavior is the result of interacting intra-personal factors (pertaining to an individual’s motivation and capability) and extra-personal conditions (associated with an individual’s environmental opportunities). Capability can be either psychological, such as knowledge, or physical, such as skills. Opportunity, on the other hand, can refer to social elements like societal influences or physical resources in the environment. Motivation may manifest in automatic forms such as emotional responses or reflective forms such as beliefs or intentions. This model thus provides an exhaustive framework for spotting behavioral patterns and their sources.

The COM-B Model is the foundational starting point of intervention development. Nested at the core of the Behavior Change Wheel (BCW), a toolkit built for the design of behavior change strategies, the components of COM-B can be charted onto the BCW^7,8^. This can then be correlated with policy categories and intervention functions, informing the development of interventions while leveraging available resources and understanding^9-11^. In theory, this framework ensures a comprehensive understanding of the elements facilitating or acting as barriers to smoking cessation in China. This could serve as a groundwork for developing appropriate interventions to promote smoking cessation.

The objective of this research was to systematically and theoretically examine the barriers and facilitators of cessation efforts among Chinese smokers by evaluating their capability, opportunity, and motivation for smoking cessation.

## METHODS

### Location and participant demographics

Semi-structured in-person interviews were conducted in Beijing, China, between April and July 2022. The sample was diversified through multiple recruitment strategies which aimed to encompass varied demographic groups and smoking behaviors. Initial participants were selected from visitors to Chaoyang Hospital’s smoking cessation clinic post-consultation. Furthermore, individuals with a smoking history from both the patient and staff demographics in Mentougou District Hospital of Traditional Chinese Medicine, were invited to contribute to our research. Community members from Xicheng District were also engaged. Furthermore, several participants were recruited from communities in Xicheng district.

Interviews were carried out in controlled, private environments, such as allocated rooms in the aforementioned medical institutions. More specifically, most interviews were conducted in a private room onsite the Chaoyang Hospital's smoking cessation clinic or interview rooms at the Mentougou District Hospital of Traditional Chinese Medicine. On occasions where such environments were unattainable, alternative locations were deemed suitable, for instance, local cafés, where participants could remain relaxed and secure.

A purposive sampling strategy was utilized to ensure a diverse participant pool. This strategy constituted four distinct groups: current smokers aged <40 years, current smokers aged ≥40 years, former smokers age <40 years, and former smokers aged ≥40 years. The research accepted both male and female participants. The inclusion of smokers and ex-smokers intended to thoroughly examine the complexities of smoking cessation, bearing in mind the differing motivations for continued smoking, quitting, relapse, and long-term abstinence, which could never be fully achieved in any single population. Participants were individuals aged ≥18 years who currently smoke or have previously smoked. Written informed consent were obtained, and participant characteristics were recorded at the initiation of the study. The interviews were distributed as follows: 5 in Chaoyang Hospital’s smoking cessation clinic, 29 at Mentougou District Hospital of Traditional Chinese Medicine, and 6 in other venues.

### Procedure

A team of five trained investigators, including XYX, TQC and BLL, who were experienced in qualitative research, conducted the interviews. At the beginning of each interview, researchers provided a concise overview of the study procedures, objectives and requested written informed consent from the participants. Prior to initiating the interviews, a brief demographic survey and a questionnaire related to smoking habits were administered to gather background information and ascertain the characteristics of the participants (Supplementary file). No prior relationship with the participants was established before the start of the study. A semi-structured interview guide (Table 1) directed the interviews, focusing mainly on the participants’ perspectives and experiences with smoking and cessation, their environmental conditions, and the available smoking cessation services. Each interview, which was audio recorded, lasted approximately 30 minutes. The interview content was transcribed into a Word document and supplemented with real-time notes by two researchers (XYX and LGZ). This study was approved by China CDC Institutional Review Board (No. 202204). The reporting of this study was compliant with the consolidated criteria for reporting qualitative research (COREQ), an endorsed checklist that promotes explicit and comprehensive reporting in qualitative studies^12^ (Supplementary file).

**Table 1:** Interview template

**Perspectives on and experiences of smoking**
When do you usually smoke?
Under what circumstances are you more likely to smoke, or smoke more? Under what circumstances are you more likely to not smoke or smoke less?
What negative effects do you think smoking has had on you?
What do you think is the advantage of smoking for you?
**Perspectives on and experiences of cessation**
Under what circumstances do you think you might decide to quit smoking?
What might encourage you to quit smoking?
What are your main concerns before you decide to quit smoking?
Please briefly describe the last and longest time you quit smoking.
What difficulties and obstacles have you encountered in quitting smoking? How do you deal with them?
What is the cause of your relapse? How does it feel to relapse?
**What kind of support do you expect for smoking cessation?**
Environment associated with smoking and cessation
How many people around you smoke?
What effect does smokers around you have on you to quit smoking?
Would you seek family support when you quit smoking? Why?
What kind of support do you think your family can give you?
**The smoking cessation services**
Have you heard about smoking cessation clinic or quit line?
Have you been to any of these places?
What do you think about smoking cessation clinic and quit line?

### Data analysis

Interviews were audio recorded and subsequently transcribed verbatim. To ensure comprehensive and accurate transcription, the written content was cross-verified with the notes made during the interviews by the researchers. Post-transcription, the transcripts were uploaded to the NVivo 11 software for further analysis, which was primarily executed by XYX and received assistance from TQC, BLL and LGZ. The analysis utilized the COM-B framework to establish a fundamental set of themes: psychological capability, physical capability, automatic motivation, reflective motivation, social opportunity, and physical opportunity. As the coding process evolved, these deductive themes underwent further refinement. In instances of coding disagreements, discussions were initiated to reach a consensus, with the intervention of a senior researcher, as required. Finally, the codes were grouped according to the respective COM-B domains. Table 2 provides a detailed overview of these codes and their corresponding categories.

**Table 2:** Summary of themes and sub-themes from the semi-structured interviews

*Themes*	*Sub-themes*
**Capability to stop smoking**	Cognitive and interpersonal skills needed to maintain smoking cessation Knowledge about smoking Knowledge about withdrawal symptoms
**Opportunity to smoking cessation**	Social climate about smoking Smoking cessation services Family support
**Motivation to stop smoking**	Beliefs about the harmfulness of smoking on own health Beliefs about the harmfulness of smoking on family Views and attitudes towards smoking Confidence in quitting

## RESULTS

### Characteristics of participants

We conducted interviews with 37 male and 3 female eligible participants, of whom 22 were former smokers and 18 currently smoked. The mean age of participants was 45 years (SD=16.2), with the average smoking duration identified as 19 years (SD=14.1). Table 3 presents detailed demographic characteristics and the smoking history of participants.

**Table 3:** Summary of participants’ demographic characteristics and smoking profiles (N=40)

*Characteristics*	*n (%)*
**Age** (years)	
18–40	20 (50.0)
40–60	11 (27.5)
>60	9 (22.5)
**Gender**	
Male	37 (92.5)
Female	3 (7.5)
**Education level**	
Middle school and lower	6 (15.0)
High school	9 (22.5)
College and higher	25 (62.5)
**Smoking status**	
Current smoker	18 (45.0)
Ex-smoker	22 (55.0)
**Duration of smoking** (years)	
<10	11 (27.5)
10–20	11 (27.5)
>20	18 (45)
**Number of quit attempts**	
0	7 (17.5)
1–3	25 (62.5)
>3	8 (20.0)

Facilitators of and barriers to smoking cessation among Chinese smokers are summarized in Table 4 and detailed below.

**Table 4:** Facilitators of and barriers to smoking cessation among Chinese smokers

*Categories*	*Main findings*
**Facilitators**	
Capability	Strong willpower was deemed as the essential capability for successful smoking cessation
Opportunity	The positive shift in societal attitudes against smoking improved smokers’ opportunity for smoking cessation
	Family members often encourage smokers to quit
Motivation	Smokers’ understanding of the health repercussions of smoking escalated when they or their acquaintances experienced serious illnesses
	Smokers’ concern for familial health, especially fetus and children significantly improved their motivation to cease smoking
**Barriers**	
Capability	Smokers often encountered difficulties in managing smoking-related temptations from peers and coping with the challenges of post-smoking cessation life after quitting smoking
	Limited understanding of smoking, cessation techniques, and withdrawal symptoms hindered smokers’ capability to quit
Opportunity	Smokers’ awareness regarding smoking cessation services was low
	Smokers’ lacked understanding about the smoking cessation services contributed to low uptake of such assistance
	The arduous and time-consuming process of seeking assistance undermined the utilization of smoking cessation services
	Familial support was under-utilized for fears of potential adverse responses from their family
Motivation	Smokers often neglect the associated risks of smoking
	Regarding smoking as a symbol of personal liberty or merely a habit deterred smokers from quitting smoking
	Over-emphasizing willpower in the cessation process thwarted smokers’ confidence to quit

### Smokers’ capability for smoking cessation

*Cognitive and interpersonal skills needed to maintain smoking cessation*

This study highlighted self-regulation and willpower as key capabilities, recognized by both current and former smokers as essential for successful smoking cessation. Nearly all participants attributed failures in quitting smoking to a lack of willpower. However, this overemphasis on willpower often resulted in them overlooking the core issues in their cessation efforts. Evidently, smokers principally struggle with managing social influences, such as invitations from others to smoke and coping with negative emotions. A significant number of smoking individuals who had attempted to quit expressed that better coping ability for these triggers could potentially have improved their cessation outcomes:

*‘I don’t think people need help to quit smoking, what is essential is their willpower.’* (Participant 10, current smoker)

*‘If you want to quit smoking, you must make up your mind and perseverance to succeed.’* (Participant 13, ex-smoker)

*‘I did not know how to reject others’ invitations properly, I was afraid that if I kept rejecting others that way, the relationship between my friends and me would be destroyed.’* (Participant 10, current smoker)

*‘The greatest difficulty in the process of quitting smoking was that my colleagues around me were inviting me to smoke.’* (Participant 18, ex-smoker)

*‘Last time I relapsed because I encountered difficulties in my life, and I was in a bad mood.’* (Participants 16 and 32, ex-smokers)

*Knowledge about smoking*

A significant number of the study participants demonstrated substandard comprehension concerning the health risks associated with smoking. In spite of an apparent self-assured knowledge regarding the harmful effects of smoking, most individuals could only identify a few health consequences, primarily lung cancer and respiratory symptoms, neglecting to acknowledge a spectrum of other smoking-related detrimental effects. Furthermore, there was a pervasive lack of understanding regarding the degree of association between these health conditions and smoking. These deficiencies in comprehension and understanding could potentially engender a shallow belief in the health consequences of smoking:

*‘Everyone says smoking is harmful, but that is all, what exactly will it cause?’* (Participant 12, current smoker)

*‘Many people do not smoke or drink in their lives, but they still suffer from cancer. So what is the relationship between smoking and cancer? It is very ambiguous for me.’* (Participant 2, current smoker)

*Knowledge about withdrawal symptoms*

The study revealed that numerous participants lacked comprehensive understanding concerning withdrawal symptoms. Although some participants had previously experienced some mild to moderate withdrawal symptoms during prior attempts to quit, the root cause of these symptoms, along with an approximate timeframe for their potential alleviation, remained unclear to them. Consequently, this lack of knowledge left them ill-prepared to anticipate and manage these symptoms in their future quit attempts. Moreover, certain participants harbored the misconception that these symptoms were indicative of severe health conditions precipitated by the sudden cessation of long-term smoking, thereby deterring them from further attempts to quit:

*‘After quitting smoking, it was not as painful as I have thought, but my eating and sleeping order was disrupted. It made me afraid.’* (Participant 12, current smoker)

*‘In fact, many people have thought about quitting smoking. But after quitting smoking, many people’s physical condition deteriorated. This is worrying.’* (Participant 17, current smoker)

### Smokers’ opportunity to smoking cessation

*Social climate for smoking*

The majority of study participants observed a gradual shift in the social atmosphere and attitudes towards smoking, noting significant changes compared to several decades ago. Many indicated the current perception of smoking, particularly in public areas, as undesirable. Some participants recounted instances of strong public condemnation of their smoking in such settings. Participants also noted a decline in the acceptance of cigarette gifting due to associated health risks, a notion considered unprecedented within China’s societal norms as recently as a few decades prior:

*‘I have talked about it with my friends, we all agreed that calls for quitting smoking will gain momentum in China, look at the no-smoking signs in public places, and how people react when you smoke, it makes me feel awkward to smoke here.’* (Participant 1, ex-smoker)

*‘The current social atmosphere is relatively better than before. Smoking used to be attractive and predominant, but now, it is an unwelcome behavior for most people.’* (Participant 6, current smoker)

*‘The social norm is turning against smoking nowadays; I used to deliver high-end cigarettes to my clients, which were deemed a fancy gift. But it is forbidden by my company now as it is believed that cigarettes are harmful to people’s health and they convey malice rather than goodwill.’* (Participant 6, current smoker)

*Smoking cessation services*

Awareness regarding smoking cessation services in China appears to be limited, as many participants indicated unfamiliarity with such resources, including smoking cessation clinics and Quitline. Some participants who had prior knowledge of these services expressed skepticism about their effectiveness, due to a lack of understanding about the difference between assisted and unassisted quitting methods, and how these services could facilitate their cessation efforts. Moreover, participants who were aware of the cessation clinics and Quitline, particularly those who had utilized them, frequently criticized the process of seeking assistance as being arduous and time-consuming:

*‘I have never heard of these [both cessation clinics and Quitline].’* (Participants 13, 16 and 32, ex-smokers)

*‘I would not turn to these [both cessation clinics and Quitline]. If I want to quit, I think what matters is my willpower. I mean, what can they do? Psychotherapy? Medication? I totally have no idea.’* (Participant 2, smoker)

*‘I have waited for the appointment for months, and this time I called for sick to see the doctor here, it is such a laborious process.’* (Participant 6, smoker)

*Family support*

The frequent encouragement from family members for smokers to quit has been widely reported. Nevertheless, a significant number of participants expressed reluctance to seek family support during their cessation journey, primarily due to the belief that smoking cessation is a personal matter not requiring outside assistance. Further, concerns were raised by some smokers regarding potential adverse responses from their family, such as criticism during episodes of relapse, as opposed to supportive engagement:

*‘My family numbers care about me, and they often urge me to quit. Every time I was found smoking, they showed disapproval. It has had a subtle effect on me.’* (Participant 30, ex-smoker)

*‘I have never discussed it with my family in my previous quit attempts, I mean, it is my own business, I cannot image what can they do to help me.’* (Participant 16, ex-smoker)

*‘You should never discuss it [quitting smoking] with your family when you are about to make a quit attempt. For example, if you told your wife you are going to quit, she may question your ability or mock you when you relapse. It makes you feel more guilty when you relapse because you feel obligated to live up to their expectation.’* (Participant 25, ex-smoker)

### The motivation for smoking cessation among smokers

*Beliefs about the harmfulness of smoking on personal health*

Numerous current smokers fall short of recognizing the severe impact smoking has on their health, often neglecting the associated risks and hence, display a lack of motivation to cease smoking. However, an array of former smokers and a minority of current smokers have conveyed that their understanding of the health repercussions of smoking escalated when they or their acquaintances experienced serious illnesses, a factor that ultimately led to their decision to stop smoking:

*‘You can’t live to 200 years old even if you don’t smoke, can you?’* (Participant 15, current smoker)

*‘This time I am determined to quit because I had lung nodules, and this resulted in the strongest impulse to quit I have ever had.’* (Participant 4, ex-smoker)

*‘It was when my sister had her stent insertion that I decided to quit, the doctor said her disease may be caused by smoking and staying up late, and we could have lost her with any delay in treatment. These [smoking and staying up late] were exactly what I were doing then, and it scared me, so I made my decision to quit that time.’* (Participant 2, current smoker)

*Beliefs about the harmfulness of smoking on family*

The majority of study participants acknowledged the potential harm smoking can inflict on their families, especially fetus and children. Their concern for familial health significantly escalated their motivation to cease smoking. In particular, the expectation of a new baby was reported to be a critical motivating factor for numerous ex-smokers for cessation and remains a serious consideration for current smokers planning to start a family. Furthermore, some current smokers reported adopting strategies to minimize the negative impacts of their smoking habits on their families. This includes self-regulation methods such as avoiding smoking at home or taking measures to disperse the produced cigarette smoke, despite not being able to quit immediately:

*‘The most important reason I quit smoking was because I worried about my family’s health.’* (Participant 1, ex-smoker)

*‘I quit smoking after my wife was pregnant.’* (Participant 12, current smoker; Participant 21, exsmoker)

*‘I guess I will quit smoking when preparing to have a baby, many people quit because of this. All my friends who have quit successfully were quitting for their babies.’* (Participant 11, current smoker)

*‘I have set up a smoking room in my house, which is separated from other rooms and is equipped with a fan to dissipate the smoke.’* (Participants 6 and 12, current smokers)

*Views and attitudes towards smoking*

Numerous smokers argued that their decision to smoke is a personal matter, reflecting their belief that as long as their habit does not negatively impact others, they maintain the right to engage in such behavior. This line of thinking provided a sense of justification for their continued smoking. Additionally, other smokers identified smoking as a habit or hobby that contributes to their life satisfaction, further reinforcing their reluctance to quit. These represented the two most frequently cited reasons behind the current smoker group’s aversion to smoking cessation:

*‘I mean, smoking is your choice and right, it deserves respect.’* (Participant 3, current smoker)

*‘In fact, that [other people’s bad impression about smoking] did not bother me at all, I only realized that smoking is unattractive after I quit smoking.’* (Participant 25, ex-smoker)

*‘I know non-smokers believe smoking is very terrible, but for us, it is just our habit, exactly the same as fishing and drinking tea.’* (Participants 10 and 12, current smokers)

*Confidence in quitting*

A considerable proportion of current smokers expressed low confidence in their ability to quit, primarily attributing successful smoking cessation to willpower. They held the conviction that only individuals with high willpower and self-discipline could achieve and maintain cessation. Consequently, for former smokers, transitioning to a non-smoking status was perceived as a commendable achievement and a source of pride. However, for current smokers, there was a prevalent fear of not being resilient enough to persevere and resist the allure of cigarettes:

*‘You have been smoking for decades, this has become a part of your life, it is definitely not easy to quit.’* (Participant 2, current smoker)

*‘It is hard to make up your mind to quit smoking especially when you have failed several times, because that the relapse will make things worse, it erodes your confidence. Some may think: since I cannot keep abstinence for sure, I would rather not to quit at all.’* (Participant 4, ex-smoker)

## DISCUSSION

This research investigated the capability, opportunity, and motivation of Chinese smokers to cease smoking, in addition to analyzing both facilitators and barriers to this process. These findings offer important insights into potential interventions for smoking cessation in China.

The primary and most apparent implication highlights the necessity for public education campaigns regarding the detrimental effects of smoking and the importance of cessation. Initially, numerous smokers perceive smoking as a symbol of personal liberty and is merely a habit. This viewpoint may partially stem from the tobacco industry’s strategy of associating smoking with an individual’s pursuit of happiness. This association can significantly influence smokers’ perspectives, often providing a plausible rationale for continuing their smoking habits^13^. It becomes imperative to implement effective campaigns aimed at persuading smokers that their autonomous decision-making is compromised when they become addicted to nicotine, thereby reducing the perceived legitimacy of the smoking habit.

While many smokers overemphasize the role of willpower in their attempts to quit, our research suggests that this mindset may inadvertently hamper their progress. Many smokers who struggle with cessation tend to broadly attribute their inability to maintain abstinence to a lack of willpower. This perspective, however, overlooks specific challenges such as managing social invitations to smoke or contending with negative emotions. By addressing these overlooked areas, their chances of maintaining their cessation efforts in the long-term may be improved. This overemphasis on willpower can also undermine smokers’ confidence in their ability to quit, falsely portraying sustained abstinence as an elusive objective attainable only by individuals with remarkable willpower. We propose that a broader educational campaign is crucial to address the prevailing misconception that smoking is a casual hobby and willpower is solely responsible for successful cessation. Instead, it should be communicated to smokers that tobacco addiction is a chronic disease requiring specialized attention and an appropriate cessation approach. This reframing of quit smoking campaigns could prompt more individuals to turn to scientific methods and smoking cessation services in their efforts to quit.

Furthermore, it is essential to encourage individuals interested in quitting to utilize available smoking cessation services. Despite China’s robust network of over 600 smoking cessation clinics^14^ and numerous regional and national quit lines, our study revealed that awareness of these services remains low, mirroring findings from a national survey^1^. The visibility of existing smoking cessation services in China must be increased to ensure more individuals are aware of these valuable resources.

Our investigation additionally presents several pragmatic implications for promoting tobacco cessation. First, it appears that personal health narratives, specifically the experience of serious illness in their acquaintances, can serve as a potent catalyst for some smokers to quit. This suggests that profiling authentic health-related consequences of smoking could resonate with smokers and potentially heighten their quit motivation – a level of motivation that they may otherwise reach only through experiencing adverse health conditions firsthand. However, foresight is always better than hindsight; it would be significantly advantageous for smokers to abandon the habit before any irreversible health consequences arise. Consequently, we posit that sharing compelling stories from affected individuals could markedly increase quit attempts, a strategy echoed in the Tips From Former Smokers (TIPS) campaign^15^. Second, there is a significant emphasis on familial health among many participants, particularly when contemplating parenthood – a finding corroborated by previous studies^16,17^. This life stage could, therefore, create a prime temporal window for tailored smoking cessation interventions. With most prospective parents actively weighing cessation at this stage, proactive engagement from healthcare institutions, promoting cessation services, could prove highly beneficial.

Furthermore, the complex and lengthy process required to obtain professional assistance may dissuade potential candidates from utilizing such services. These could partly explain the low usage rate, as nearly 90% of smokers who attempted to quit over the course of the previous year did not seek professional guidance^18^. Therefore, it is critical to enhance the accessibility of smoking cessation services, taking inconvenience complaints into account. A promising solution could be a smartphone smoking cessation application. This technology has the potential to offer a readily accessible, cost-effective, and efficient method to facilitate smoking cessation^19^ and has been ardently welcomed by a significant number of Chinese smokers^9,20^.

Enhancing family support is crucial in assisting smokers to attain sustained abstinence. In China, the primary focus of smoking cessation interventions has largely been on smokers themselves, from increasing their motivation to augmenting their problem-solving abilities. This facet of intervention is undeniably essential. However, the role of a smoker’s family should not be neglected. Generally, family members desire the best outcome for the smokers and aim to aid them in quitting. Yet, this potential source of support is often underutilized or inadvertently obstructive within most smokers’ households. We strongly advocate for incorporating family education in smoking cessation interventions to aid family numbers in comprehending the challenges and difficulties associated with smoking cessation and guide them in how to support a quit attempt constructively. This could be fundamental in helping quitters to maintain abstinence.

Our study also underscores the significance of advocacy campaigns aimed at shaping social norms surrounding smoking. In the late 20th century, research often found that cigarette smoking was viewed both as an accepted social activity and an integral part of social interaction, notably among adult males^21,22^. Multiple qualitative studies^16,23-25^ emphasized the influence of traditional culture in relation to smoking, with smokers frequently experiencing pressures related to cigarette sharing and gifting customs. Moreover, prevalent misperceptions of smoking exist, such as the symbolization of masculinity. Presently, smoking in public spaces has become more socially unwelcome. Negative remarks can be prompted, and gifting cigarettes is perceived as improper by some individuals. In other words, Chinese society does not advocate for smoking to the same extent as it did decades ago. During this era, advocacy campaigns like ‘Giving Cigarettes is Giving Harm’^26^ may have played a crucial role. Further sustained investments in such campaigns hold the potential to mold the social norm towards a more anti-smoking stance.

Findings from the ITC China Survey^27^ indicate that concern for personal health, as well as the health of friends and family members, markedly influences smokers’ intent to quit. However, there is a complex dynamic presented among Chinese smokers. Despite the pervasive knowledge of the detrimental effects of smoking, many smokers do not fully acknowledge these health risks unless they are personally afflicted with severe ailments. Interestingly, the same individuals demonstrate a heightened awareness regarding the health consequences that smoking poses to their family members. A notable segment of smokers, in response to this understanding, engage in practices intended to mitigate the harm inflicted on their families; such actions include reducing the amount smoked indoors and actively diverting smoke from within the household. Future research should be dedicated to investigating this complex behavior.

### Strengths and limitations

The primary advantage of this research is the utilization of the COM-B as a deductive tool, providing a comprehensive insight into smoking trends and cessation in China. This approach facilitated a thorough evaluation of the factors that promote and obstruct smoking cessation among Chinese smokers. Moreover, participants were categorized based on age and smoking status constraints, ensuring data diversity. In addition, the inclusion of several female smokers in the participant pool furthered data heterogeneity.

The generalizability of the findings, however, might be limited due to the relatively small sample size and potential selection bias. Notably, the majority of the participants were recruited from medical institutions and possessed a high level of education. The use of convenience sampling in selecting study locations and participants may have led to a sample that may not be representative of the broader Chinese population. Furthermore, the structured format of the study, grounded in theory, could have possibly constrained participants’ responses.

## CONCLUSIONS

This study employs a theory-based qualitative method to comprehensively examine the facilitators and barriers that Chinese smokers perceive to smoking cessation. Smokers in China could further benefit from targeted interventions that duly consider COM-B factors that either inhibit or encourage cessation. Educational and publicity campaigns should aim to debunk smokers’ misperception that smoking is a symbol of personal freedom and rectify their undue reliance on willpower. It is also suggested to promote currently available smoking cessation services more broadly and socially depreciate the appeal of smoking. Strategic approaches including sharing real-life accounts of the health ramifications of smoking, providing information about smoking cessation services at maternity centers, expanding the existing smoking cessation services by integrating mobile health, and empowering families to support smokers in their quit attempts, may significantly facilitate smoking cessation in China.

## Data Availability

The raw data (interview transcript) that support the findings of this study are available from the corresponding author upon reasonable request. The interview records and transcribed documents cannot be made available for privacy reasons.
